# Heterogeneous Perfusion in COVID-19 and High Altitude Pulmonary Edema: A Review of Two Cases Followed by Implications for Hypoxic Pulmonary Vasoconstriction, Thrombosis Development, Ventilation Perfusion Mismatch and Emergence of Treatment Approaches

**DOI:** 10.7759/cureus.10230

**Published:** 2020-09-03

**Authors:** Isaac Solaimanzadeh

**Affiliations:** 1 Internal Medicine, Interfaith Medical Center, Brooklyn, USA

**Keywords:** covid-19, sars-cov-2 (severe acute respiratory syndrome coronavirus -2), high altitude pulmonary edema, ventilation perfusion mismatch, pulmonary vasodilation, covid-19 respiratory failure, hypoxic injury, hypoxic pulmonary vasoconstriction, covid-19 management, coronavirus disease 2019 (covid-19)

## Abstract

Coronavirus disease 2019 (COVID-19) has been compared to high altitude pulmonary edema (HAPE). Multiple similarities between the two conditions were drawn in the past. This article seeks to further clarify potential underlying mechanisms related to hypoxia and pulmonary vascular responses. It does so by looking at perfusion imaging of patients with COVID-19 and comparing them with patterns observed in HAPE and hypoxic exposure.

Two separate clinical cases are reviewed. The salient aspect of each case that is emphasized is the perfusion scintigraphy results that revealed heterogeneous perfusion patterns in both patients. Heterogeneous or non-homogeneous perfusion is also observed in HAPE. A detailed clinical course of each patient is described. Medications utilized to treat the conditions are outlined as well as laboratory parameters and clinical findings. Interestingly, both of these patients were treated with calcium channel blockers and this class of medications is utilized to prevent HAPE as well. Discussion following the case presentations attempts to contextualize possible implications of this and other studies on the broader pathophysiology of COVID-19 disease.

Findings related to pathophysiologic patterns and treatment strategies are also described. Micro-thrombi formation has been reported in both COVID-19 and HAPE as well and may be an accessory complication of perfusion compromise. In a separate study, vasodilatation with calcium channel blocker (CCB) therapy has been associated with improved mortality in COVID-19 and potential pathophysiologic mechanisms were previously presented. This case report provides further clinical findings that support the notion that perfusion deficits are an integral component of hypoxia in COVID-19. It also advances the basis for use of vasodilator therapy as part of treatment regimens in COVID-19. Vasodilators may improve micro-perfusion. In this way, oxygenation may be promoted by decreasing impedance and improving flow via the alveolar-capillary unit.

## Introduction

Hypoxia is a central aspect of both COVID-19 and high altitude pulmonary edema (HAPE), even though they are each separate entities and emanate from distinct triggers. Nevertheless, counteracting similar pathophysiologic trends may be essential for the improvement of both conditions. This article explores the relationship between perfusion patterns in these two conditions. It also seeks to elaborate on what may be happening as part of relatable disease processes. Finally this article culminates with counteractive treatment strategies that follow pertinent clinical findings.

To begin with, heterogeneous or uneven or non-homogeneous hypoxic pulmonary vasoconstriction (each term is used herein interchangeably) has been suggested to be a general phenomenon in animals and humans alike. This uneven pulmonary perfusion has been observed in humans exposed to hypoxia and is more pronounced in individuals susceptible to HAPE [1]. Non-homogeneous hypoxic pulmonary vasoconstriction has been observed in rats exposed to hypoxia as well suggesting that hypoxia induces heterogeneous pulmonary blood flow distribution which is followed by increased vascular permeability and the development of pulmonary edema [2].

This was also demonstrated with exposure to hypoxia. Functional magnetic resonance imaging in HAPE-susceptible individuals exposed to acute hypoxia exhibited increased pulmonary blood flow heterogeneity, consistent with uneven hypoxic pulmonary vasoconstriction [3]. Altogether, hypoxic exposure plays an integral role in the development of a heterogeneous pulmonary perfusion pattern. This has important implications for treatment of HAPE as well as for COVID-19.

Consider that in HAPE, the dihydropyridine calcium channel blocker (CCB) nifedipine is an effective medication for preventative treatment [4].

In tandem, in COVID-19, dihydropyridine calcium channel blockers nifedipine and amlodipine were found to be associated with significantly improved mortality survival and decreased risk for intubation in elderly patients hospitalized with the acute viral illness [5].

How might CCBs work to improve outcomes in both HAPE and COVID-19? Vasodilatation may allow for improved perfusion. The heterogeneous vasoconstriction is reversed and perfusion thereby ensues. Increased regional pulmonary capillary pressures accompanied by stress failure may be a result of uneven hypoxic pulmonary vasoconstriction [6]. Vasodilators may function to relieve this. Micro-perfusion or flow via the alveolar capillary unit is subsequently allowed to resume and oxygenation may thereby improve. Capillary injury, edema, and inflammation may all be mitigated as well.

Two separate clinical cases will be reviewed. The aspect of each case that is emphasized is the perfusion scan finding of heterogeneous perfusion in both. These results in addition to the CCB medications that were administered in both patients are highlighted. Discussion following the case presentations attempts to contextualize possible implications on the pathophysiology of COVID-19 disease. Please note that in the interest of clarity and ease for review, the various tables outlining information related to both Patient A and B were combined. However, the descriptive clinical course of each patient is separate and demarcated.

## Case presentation

Patient A

Patient A is a 59-year-old female with a past medical history of obesity, hypertension, cholelithiasis, and gastritis. She is also a former smoker (quit several years prior) that presented following a one-week history of loss of appetite and generalized weakness. The patient's daughter tested positive for COVID one week prior to presentation and had been visiting the patient who lives with other family members including her grandchildren.

Emergency medical services reported an oxygen saturation level of 74% on initial encounter and prior to administration of oxygen via a non-rebreather. It is intriguing that the patient denied any shortness of breath. She also denied any chest pain, fever, rhinorrhea, body aches, chills, rigors, or cough.

Clinical findings upon admission

Triage vitals are outlined below in Table 1.

**Table 1:** Vital signs of Patients A and B respectively collected on admission day 1. T: Temperature Fahrenheit; HR: Heart Rate; RR: Respiratory Rate; BP: Blood Pressure; Sat: Saturation.

	T	HR	RR	BP	Sat	Oxygen Supplementation
Patient A	99.5	128	18	111/65	84%	15L Non-Rebreather
Patient B	97.7	79	26	112/75	95%	5L Nasal Cannula

Initial placement of non-rebreather helped the patient saturate up to 92% that improved further to 96% with prone positioning. However later on, saturation levels decreased and the patient required high flow oxygen - up to 30L to achieve a saturation level above 90%. Electrocardiogram was unremarkable aside from sinus tachycardia. Several laboratory parameters were elevated (see Table 2).

**Table 2:** Laboratory findings for Patients A and B. LDH: Lactate dehydrogenase; CRP: C-reactive protein; IL-6: Interleukin 6; ESR: Erythrocyte sedimentation rate; ng/mL: nanograms per milliliter; U/L: international units per liter; mg/L: milligrams per liter; pg/mL: pictograms per milliliter; mm/hr: milliliters per hour.

Laboratory	Patient A	Patient B	Reference
D-Dimer	2609	1739	0-500 ng/mL
LDH	619	930	125-220 U/L
CRP	105	121	0-10 mg/L
IL-6	38.7	Not Done	0-15.5 pg/mL
ESR	94	>120	0-22 mm/hr

**Figure 1:**
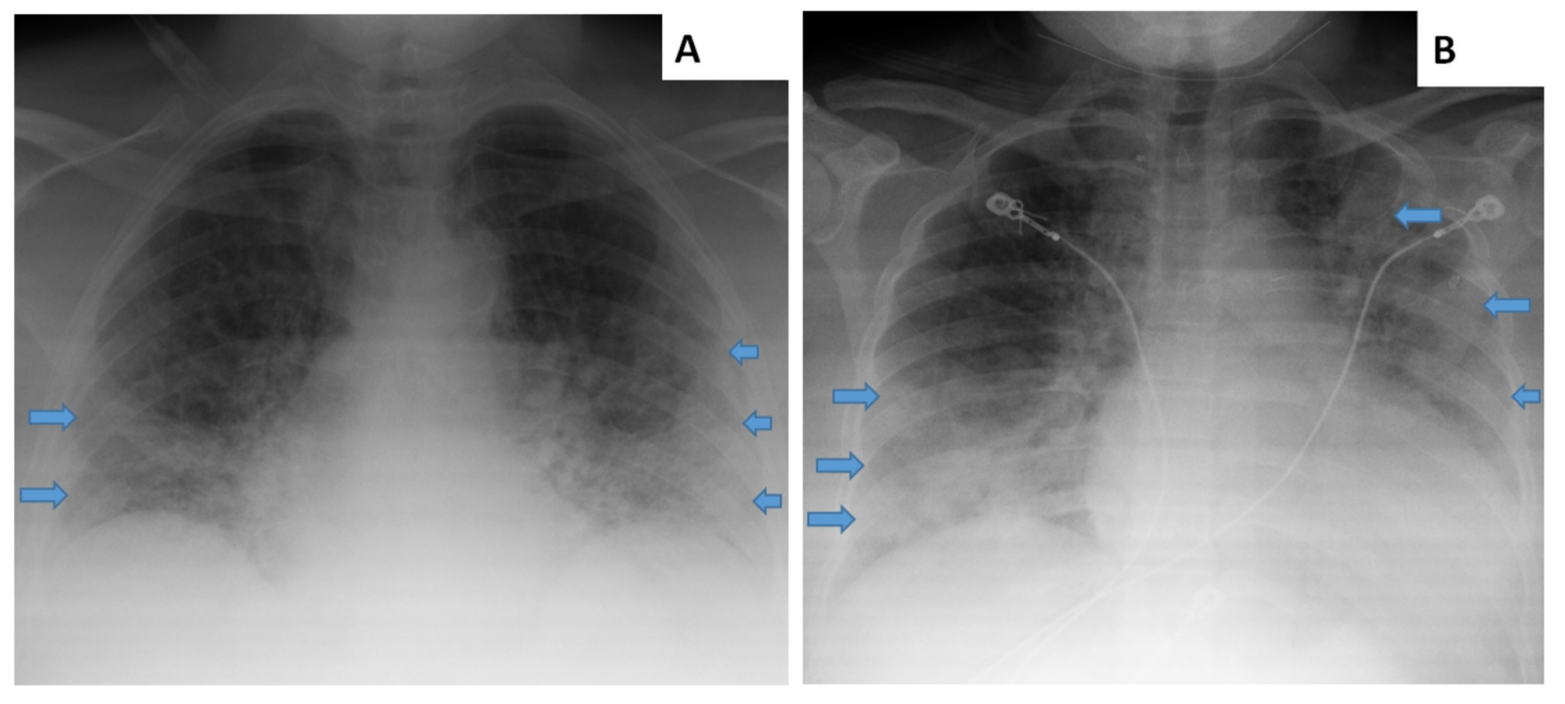
Chest X-rays of Patients A and B. Arrows indicate some portions of affected regions.

Chest X-ray upon admission, as read by a radiologist, noted that: “Pulmonary vascularity is mildly enlarged. There is diffuse increase in interstitial markings. There are bilateral lung infiltrates and perhaps some ground-glass densities peripherally on the left. No pleural effusion or pneumothorax...constellation of pulmonary findings…which have been reported in COVID-19 pneumonia…no pleural effusion” (see Figure 1).

Upon admission, the patient was started on broad spectrum antibiotics, steroids, anti-coagulation, as well as anti-viral therapy for a five-day course (see Table 3). Acute kidney Injury was also appreciated with a glomerular filtration rate (GFR) of 39.9 ml/min upon admission. Three months prior, the patient’s GFR was 69.9 ml/min.

**Table 3:** Medication treatment regimens for Patients A and B. BID: Twice daily; mg: milligram; SC: Subcutaneous; ml: milliliter; Q12hr: Every twelve hours; Q8hr: Every 8 hours; gm: grams; IVPB: Intravenous piggyback; D5: 5% Dextrose; ml/hr: milliliters per hour.

	Patient A	Patient B
Anti-coagulation	Enoxaparin 150 mg SC daily (later increased to 140 mg q12 hr)	Enoxaparin 40 mg SC daily
Steroid	Methylprednisolone 40 mg IVPB Q12 hr	Methylprednisolone 40 mg IVP Q8 hr
Antibiotics	Ceftriaxone 1 gm IVPB daily	Ceftriaxone 1 gm IVPB daily
	Azithromycin 500 mg IVPB daily	Doxycycline 100 mg PO BID
Fluid	1/2 Normal Saline 100 ml/hr	D5 1/2 NS 100 ml/hr
Anti-viral	Hydroxychloroquine 400 mg PO BID (after loading dose, continued dose at once daily)	Hydroxychloroquine 200 mg PO BID
Other	Pepcid 20 mg PO BID	
Anti-hypertensive (added later); also vasodilators	Amlodipine 5 mg (later increased to 10 mg) PO daily	Nifedipine 30 mg extended release (later increased to 60 mg) PO daily

**Figure 2:**
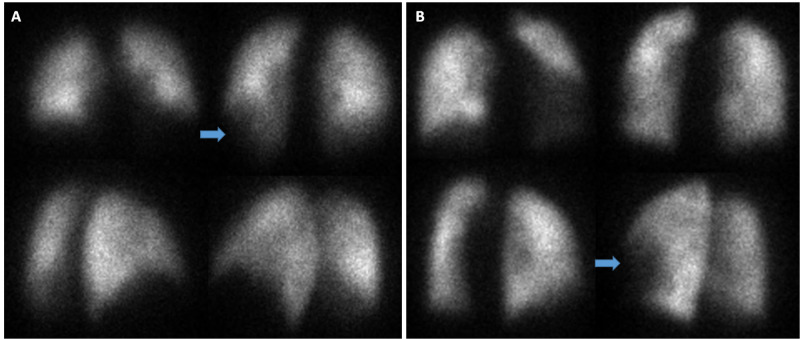
Perfusion scintigraphy for Patients A and B. Although segmental defects are indicated with arrows, the key take-home point or revelation lies in the overall *perfusion pattern *being one that is *heterogeneous *and not homogeneous. This reflects an abnormal state that is multi-lobular and one that is posited to reflect compromised perfusion in this hypoxic condition.

On day 2, a perfusion scan was conducted to rule out pulmonary embolism (Figure 2). Results were notable for an “abnormal ventilation/perfusion lung scan classified as intermediate probability for pulmonary embolism.” Yet, the overall pattern of perfusion distribution is an important finding in its own right and that is a focus of this article that will be further elaborated below. The radiologist observed the following:

“*There is **Nonhomogeneous distribution **of the radiotracer noted in the lungs bilaterally on perfusion images. There is a large segmental defect noted in the left lower lobe (superior and inferior segments; more prominent anteriorly). There is also small segmental defect seen in the right lower lobe. All above described defects appear corresponding to the infiltrate seen in the chest X-ray of the same region.”*

Given intermediate probability as well as clinical suspicion anticoagulation with enoxaparin was increased to 140 mg q12 hours.

On day 3 of hospitalization, amlodipine 5 mg PO daily was added given elevated blood pressure. Parenthetically, this is another important aspect of the clinical case related to vasodilation that will be discussed further below. Furthermore, both patients were previously prescribed calcium channel blockers as part of treatment for hypertension prior to hospitalization.

On day 5, the patient denied any new complaints. She denied any shortness of breath, chest pain, cough, or fever. Although the medical notation did report that she appeared mildly anxious. Saturation level was 94% on high flow oxygen, but now at a higher rate than before; 60 L/min FiO2: 50%. The patient was also observed to de-saturate dramatically when off the oxygen.

Laboratory testing for SARS-CoV-2 polymerase chain reaction (PCR) returned positive. Blood cultures were negative. Renal function returned to baseline with intravenous hydration. A CT angiogram was pursued on day 7 that showed no evidence of a large central pulmonary embolism, however less than ideal test results made evaluation of segmental and sub-segmental pulmonary arteries extremely limited. Therapeutic dosage of Enoxaparin was nonetheless continued. Also, on day 8, amlodipine was increased from 5 to 10 mg daily given elevated blood pressure.

Gradually oxygen saturation levels improved and less oxygen supplementation was required. Although saturation levels had improved, de-saturation was still apparent without oxygen and with walk testing. The patient was nonetheless downgraded from the more acute care stepdown unit to the floor unit on day 11.

**Figure 3:**
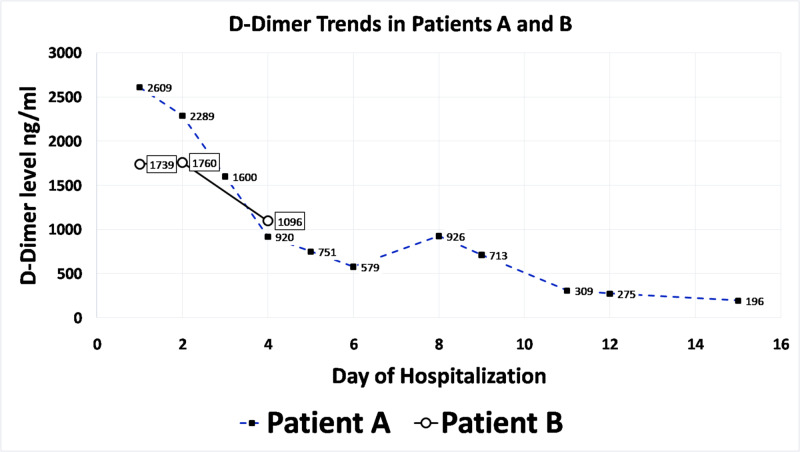
D-Dimer Trends in Patients A and B.

Concurrently, D-Dimer levels also consistently declined (Figure 3).

The Enoxaparin dose was later reduced. Notably, the patient reported vaginal spotting on day 13, at which point the Enoxaparin dose was discontinued. Thereafter, vaginal spotting ceased. Outpatient follow-up with gynecology was arranged for extensive workup. Enoxaparin was resumed a couple of days later but at a lower prophylactic dose of 40 mg SC daily.

Finally, the patient's saturation levels improved to the point where she was able to saturate at >94% on room air for more than 72 hours. Upon discharge, on day 17, a short course of prednisone and anticoagulation was continued as well as amlodipine for hypertension (Table 4).

**Table 4:** Discharge medications. Some of the medications prescribed on discharge. Other medications not listed here include laxatives, acetaminophen, and a multivitamin. Notice a shared theme of medication classes: 1) Anti-hypertensive that is also a vasodilator. 2) Anti-coagulation, and 3) Steroid. Even though each was given for different reasons, concomitant benefits of these three medications classes may yield a triple bundle in the treatment of COVID-19 as well. But this is a separate aspect that is discussed below as well as in a prior article [5].

Patient A	Patient B
Amlodipine 5 mg PO Daily	Nifedipine 60 mg PO Daily
Rivaroxaban 20 mg PO Daily for 14 days	Aspirin 81 mg PO Daily
Prednisone 15 days taper (20 mg x 5 days, 10 mg x 5 days, 5 mg x 5 days)	Prednisone 20 mg PO Daily for 5 days
Famotidine 20 mg PO Daily	Hydroxychloroquine 200 mg PO BID
	Sodium Bicarbonate 650 mg PO TID
	Ferrous Sulfate 325 mg PO TID
	Doxercalciferol 1 mcg PO Daily

Patient B

Patient B is a 35-year-old female with a past medical history of systemic lupus erythematosus (SLE; reportedly diagnosed in 2001), hypertension, chronic kidney disease and cerebral vascular accident (CVA) with no residual weakness. She arrived in the emergency department (ED) complaining of shortness of breath. This was associated with generalized body aches, as well as subjective fevers for the prior two days. The patient had visited her primary doctor before being sent to the ED. On arrival the oxygen saturation level was 85% on room air but improved with oxygen supplementation of 5 L via nasal cannula. The patient denied any cough, chest pain, palpitations, nausea, vomiting, or change in bowel and urinary habits.

Upon admission vitals are detailed in Table 1. Laboratory findings were notable for elevated levels of D-Dimer, lactate dehydrogenase, C-reactive protein, Interleukin-6, and erythrocyte sedimentation rate (see Table 2).

Also, hemoglobin level upon admission was 8.8 g/dl and this was below the patient’s prior baseline of 10.1 g/dl approximately three months prior. In addition, the GFR was reduced to 7.79 ml/min (Blood urea nitrogen (BUN): Creatinine (Cr) = 66.4 mg/dL: 6.46 mg/dL) from three months prior when it was 13.62 ml/min (BUN:Cr = 98.0 mg/dL: 3.95 mg/dL). The bicarbonate level at the time was 15 mEq/L (normal 22-29) and potassium was 4.2 mmol/L (normal 3.5-5.1).

Chest X-ray upon admission revealed the following as interpreted by a radiologist: “bilateral airspace disease, left greater than right which could be due to pneumonia. This finding can be seen in patients with COVID 19.” (see Figure 1).

She was admitted to the stepdown unit for management of pneumonia to rule out COVID-19 complicated by acute on chronic kidney disease. Empiric antibiotics, intravenous fluids, steroids and anticoagulation were all started upon admission (see Table 3).

Interestingly, given the patient’s history of SLE, she had already been prescribed hydroxychloroquine 200 mg PO BID and this was continued in hospital.

Pulmonary embolism was considered initially and a ventilation perfusion scan was ordered as part of the workup (see Figure 2). The results were “classified as intermediate to low probability for pulmonary embolism.” However, the pertinent aspect of the perfusion scan highlighted in this article is the following description on the part of the radiologist reviewing the study:

“There is **heterogeneous distribution** of the radiotracer noted in the lungs bilaterally on perfusion images with partially segmental perfusion defects noted in the left upper and lower lobe and right middle lobe…Current chest x-ray demonstrates bilateral patchy infiltrates.”

Again, this is a heterogeneous or non-homogeneous pattern that is consistent with results in Patient A, further discussion on this to follow.

The patient’s blood pressure was not elevated upon admission. However, on the second day of admission, nifedipine extended release 30 mg PO Daily was started as blood pressure consistently increased and even reached as high as 162/112 mmHg. On the third day, the nifedipine dose was increased to 60 mg PO Daily and this was continued throughout the hospitalization.

Rheumatology and nephrology consultations were appreciated. Management suggestions were incorporated into care. Additional laboratory testing was sent. Results were not revealing of an acute SLE flair (see Table 5).

**Table 5:** Patient B laboratory results related to anemia and systemic lupus erythematosus (SLE). L*: Low; H*: High; AI: Immunoassay; ug/dL: micrograms per deciliter; ng/mL: nanograms per milliliter; IU/mL: international units per milliliter; mg/dL: milligrams per deciliter; U/mL: units per milliliter; Ab: antibody.

Laboratory Test	Value	Reference : Units
Iron Level	14 L*	27-159 ug/dL
Total Iron Binding Capacity	170 L*	250-450 ug/dL
Iron Saturation	8 L*	15-55%
Unsaturated Iron Binding Capacity	156	131-425 ug/dL
Ferritin	373 H*	15-150 ng/mL
Smith IgG Ab, Qual	<0.2	0.0-0.9 AI
Double Strand DNA Ab	4	0-9 IU/mL
Anti-Nucleolar Antibody	Positive	Negative
Complement C3	146	82-167 mg/dL
Complement C4	42	14-44 mg/dL
Total Complement (CH50)	>60	>41 U/mL

Discussion between the primary medical team and respective consultations in assessment of the overall clinical condition led them to continue methylprednisolone and hydroxychloroquine treatment but mycophenolate was not recommended. Nephrology recommended intravenous hydration as well as bicarbonate and later doxercalciferol supplementation.

The patient’s condition improved and she was downgraded to the medical floor. Oxygen supplementation requirements decreased gradually and the patient was weaned off nasal cannula. Renal function improved consistently during the hospitalization. The final creatinine level was 3.95, GFR 13.74, and bicarbonate 23 mEq/L. Steroid dosage was also decreased. An iron panel suggested iron deficiency anemia given low serum iron and iron saturation levels, despite high ferritin levels. The latter may be a result of an acute inflammatory response. The clinical team recommended iron supplementation and planned for outpatient follow-up. The patient finished the antibiotic course and maintained stable oxygen saturation levels on room air.

Patient B did have negative results for SARS-CoV-2 PCR testing twice. However, this is not an isolated case and there have been many patients that presented with typical clinical signs and symptoms consistent with COVID-19, during the time period when the outbreak was ongoing, as well as within the vicinity of this hospital and have nonetheless tested negative. Many of these patients were suspected to have false negative tests. Sensitivity of testing is called into question and negative nasopharyngeal and oropharyngeal swabs do not necessarily rule out infection [7]. Also, imaging tests have in some cases been found to be more accurate than laboratory testing [8]. Therefore, this patient is still suspect of having had and been treated for COVID-19. Antibody testing was not readily available at the time of hospitalization. The primary clinical team made the following notation in their assessment: “Despite COVID test negative, patient’s clinical features and Chest X-ray suggestive of COVID pneumonia.” The reader is welcome to form their own opinion with the information presented.

Discharge occurred on day 9 and highlighted medication regimens prescribed for continued therapy as outpatient are included in Table 4. Aspirin was continued given history of prior CVA. Hydroxychloroquine was continued given the history of SLE. Follow-ups with rheumatology and nephrology specialty clinics were recommended within 2-4 weeks as well as follow-up with a primary care physician within one week.

## Discussion

Although each clinical case presented may be interesting in their own right, the focus of this article is on the perfusion scintigraphic results. Specifically, the heterogeneous perfusion (aka non-homogeneous or inhomogeneous) pattern as this may be revealing of an underlying pathophysiologic process. This is the salient feature that is highlighted herein. The heterogeneous distribution of the radiotracer is reflective of non-homogeneous or heterogeneous perfusion. This is consistent with findings in hypoxic pulmonary vasoconstriction described above. Treatment with calcium channel blockers and other vasodilators may counteract this as is conducted in HAPE. Non-homogeneous perfusion observed in these two patients suffering from COVID-19 is reflective of increased pulmonary blood flow heterogeneity in acute hypoxia, and this is consistent with uneven hypoxic pulmonary vasoconstriction in HAPE-susceptible individuals exposed to hypoxia [3].

In step with constriction is coagulation formation. So long as the reader is accepting of Virchow’s triad. This may be viewed as part and parcel of a continuum of disease wherein constriction begets decreased blood flow and ultimately clot formation. This has been previously described in further detail as a potentially five-step progressive process considering the virally provoked inflammation, immune response and hypercoagulability as well in the milieu of COVID-19 disease [5]. Increased clot formation in COVID-19 is consistently observed. However, clot formation in HAPE may not be expressed in many patients presenting in today’s current clinical practice since effective precautionary measures may stave off advanced disease and clot development. Measures such as rapid descent from altitude, oxygen supplementation, or even medical therapies may be immediately implemented. HAPE is a well-recognized entity that has been characterized and studied. Mountain climbers and their guides are aware of various challenges, risks, and dangers that accompany ascent to high altitude. Therefore, when symptoms emerge, quick corrective actions are pursued and sequelae of disease progression are efficiently deterred, prevented or circumvented. However, this may have not been the case prior to current widespread awareness of this condition and its preventative treatment approaches. Actually, in the not so distant past, a thorough pathology examination was conducted to further investigate pulmonary changes that occur in patients that died of HAPE [9]. Herein a salient descriptive summary is worth quoting:

“*Striking changes were observed in the lung. In addition to edema and congestion which were universally present, a significant number showed intra-alveolar fibrin, hemorrhage, hyaline membranes and thrombi in alveolar capillaries. In a few cases one or more of the latter were predominant microscopic findings. In one case vascular thrombi were also observed in the kidneys and liver.”*

A recent study compared patients that died from COVID-19 with others that died of acute respiratory distress syndrome (ARDS) secondary to influenza [10]. This study shed light on the specific impact that a compromise of micro-perfusion of alveolar capillaries may have therein as well. Here pulmonary vessels in patients with Covid-19 showed widespread thrombosis with microangiopathy and alveolar capillary microthrombi were nine times more prevalent. Distinctive vascular features consisting of severe endothelial injury associated with the presence of intracellular virus were also observed. Angiogenesis was also underscored as occurring more than twice as likely in COVID-19 patients.

Angiogenesis reflects an adaptive response to tissue hypoxia, which occurs under a wide variety of situations ranging from embryonic development to tumor growth and is generally dependent on the accumulation of hypoxia inducible factors [11]. Angiogenic-gene expression was also identified in COVID-19 patients [10]. Ultimately, microthrombi and endogenous mechanisms in response to tissue hypoxia are apparent in COVID-19 as well. This may be illustrated by observing microthrombi captured on separate pathologic specimens of HAPE and COVID-19 [9,12].

These features are not dissimilar from HAPE as described above. In HAPE, vasodilatation with nifedipine has been effective for preventive treatment of disease [4]. Vasodilation may allow for improved flow (aka micro-perfusion). With improved flow there may be decreased micro-thrombi development as well as improved oxygenation.

**A hyper-focus on ventilation to improve hypoxemia is one sided. Hypoxemia may not be simply a result of poor ventilation. It can equally be a part of the lack of adequate perfusion even to well ventilated regions. If perfusion is compromised, the oxygen carrying capacity may be severely limited.** In reality, improved lung aeration following an increase in positive end-expiratory pressure (PEEP) in ARDS is not always consistent with reduced shunt and VQ mismatch. Since poorly matched redistribution of ventilation and perfusion may explain detrimental changes in shunt and VQ mismatch despite improved aeration [13]. This was also demonstrated in a study that examined lung recruitability in COVID-19 patients wherein a majority of patients were poorly recruitable with high PEEP [14]. Aeration and ventilation may not be as important in COVID-19 as is perfusion and micro-perfusion. Improved micro-perfusion may improve oxygenation, decrease clot formation, as well as expedite clearance of fluid accumulation associated with and recruited by marked inflammation.

In HAPE susceptible patients, hypoxia induced redistribution of pulmonary blood flow without a shift in ventilation [15]. Even so, vasodilatation with medications such as nifedipine is beneficial in HAPE and improved oxygenation is achieved. Perfusion improvement via decreasing hypoxic pulmonary vasoconstriction, VQ mismatch and shunting may be responsible for ameliorating hypoxemia therein. So too, COVID-19 can improve via the same mechanism. It may be argued that intravascular vasodilation agents are more desirable to bypass diffusion barriers faced via inhalation. As the latter may proscribe reach into areas that stand to improve most from vasodilation. This is coming from the perspective that regions succumbed to mild ventilation impediments by diffusion barriers given edema and inflammation collections are perhaps the preferred therapeutic targets when a bilateral *widespread diffuse *hypoxic process is in effect.

Questioners may ask: Might that induce worse ventilation perfusion mismatch since now perfusion would be promoted to poorly ventilated regions? Sure, if a specific limited locale of the lung exhibited compromised ventilation. However, when a diffuse bilateral process is in place, the overall hypoxic pulmonary vasoconstrictive response may be aberrant and counterproductive as described before. Therefore, alleviating this quandary with indiscriminate perfusion promotion throughout the bilateral pulmonary vasculature can yield an overall net effect of improve oxygenation as a result. A better response to the question posed at the beginning of this paragraph is: How else would one explain improved outcomes with vasodilation in HAPE? As mentioned in the introduction, uneven hypoxic pulmonary vasoconstriction, as well as, increased vascular resistance across the vascular bed is reflective of the same phenomenon and both benefit from vasodilation therapy. Certainly there is much more on this topic already published. However, considering this information in relation to COVID-19 disease may prove beneficial. Yet, intravascular vasodilation need not be the sole option though, as inhalation of vasodilation agents can also achieve significant distribution and penetrate bilateral diffuse lung fields. However, in COVID-19 treatment of the virally provoked endothelial inflammation may potentially be more efficacious if delivered intravascularly.

Take into account that the all too often repeated progressively deleterious trajectory of increasing oxygen supplementation via nasal cannula, non-rebreather, high-flow, followed by non-invasive and then finally invasive ventilation may provide additional oxygen needs only temporarily. When viewed from an overall perspective, adjusting fractional oxygen as well as positive end expiratory pressure are also final links to a deteriorating clinical course. **Yet, with an ongoing progressive perfusion deficit, no amount of increasing ventilatory support can overcome an intrinsic inability to perfuse the alveolar capillary unit.** Lack of adequate perfusion will not render well ventilated alveoli capable of sufficiently oxygenating hemoglobin carrying red blood cells. This is not to mention diffusion compromise as a result of fluid accumulation and edema, much of which contains inflammatory-related cellular contents. Altogether, reframing our focus on perfusion optimization rather than reactive ventilation countermeasures may yield better outcomes.

Viral injury and provocation induce significant inflammation that often is associated with concomitant proteinaceous fluid accumulation. Regardless of fluid accrual, diffusion of oxygen along the inflamed endovascular lining of the alveolar-capillary unit is compromised. Ensuing hypoxemia can be followed by hypoxic pulmonary vasoconstriction - that may be uneven and heterogeneous reflecting heteronomous endovascular sites of viral affliction. Thus, further perfusion deficits emerge and ultimately a vicious cycle that fosters thrombosis development is set in motion.

Considering that viral injury and provocation bears an assault in a diffuse pattern in the bilateral multi-lobar lung fields, it is not difficult to reckon that this formidable challenge precipitates increasing levels of hypoxia, hypoxemia, and respiratory failure. This intravascular process and pathophysiologic sequence severely limit the therapeutic suitability and enduring benefit of ventilatory support, as the deficit lies not in ventilation. Rather pathological impairment emanates primarily from the compromised diffusion of oxygen, consequent vasoconstriction and perfusion impedance along the microvascular strata of the alveolar capillary unit.

The increasing acceptance of including anti-coagulation in patients hospitalized with COVID-19 is, in essence, a measure that aims not only to avoid thrombosis formation but concomitantly maintain perfusion. The two are essentially one in the same. Ideal perfusion cannot be maintained if thrombosis exists. However, with the increasing awareness that micro-clots exist in COVID-19 benefits of anti-coagulation extend beyond classic embolism formation. Anti-coagulation therefore also promotes perfusion via the treatment or prevention of micro-clots. Elucidating broader benefits and rationale of anti-coagulation can guide us towards a rational correlate of improving perfusion: vasodilation.

Vasodilation may function to recruit further functional perfusion. Similar to the recruiting of poorly ventilated regions of lung with changes in ventilation parameters such as PEEP, improvement of otherwise poorly perfused oxygenation junctions at alveolar-capillary units may be achieved with vasodilation. Certainly, other compromisers of perfusion such as thrombosis, or inflammatory mediators are contributors and may be curtailed with anti-coagulants and steroids, respectively. However, vasodilation need not be viewed as a dangerous proposal that may undermine physiologic mechanisms to preserve effective oxygenation via hypoxic pulmonary vasoconstriction. Since, that physiologic response may be aberrant and not prone for beneficial advantage in hypoxia associated with COVID-19, similar to HAPE. Just as vasodilation is desirable in HAPE wherein a heterogeneous perfusion pattern is present, so too vasodilation may be essential for mitigation of COVID-19 wherein a heterogeneous perfusion pattern can also exist. The heterogeneous patterns in these cases support this. Moreover, clinical data from a retrospective review revealing an association with improved mortality supports this as well [5].

Hypoxic pulmonary vasoconstriction is an innate mechanism integral for fetal circulation. It may also function as a safety mechanism to promote and maintain oxygenation for preferential perfusion of well-ventilated regions of the lung. However, in an overwhelming hypoxic circumstance perhaps it may be counterproductive. It is not unheard of that homeostatic or reactive physiologic mechanisms are capable of morphing into unwitting accomplices of a vicious chain reaction. Consider for example, left ventricular hypertrophy development in chronic untreated hypertension. Although responding in an expected manner to maintain circulation, its development can nonetheless portend deleterious consequences in the long term. Therefore, counteractive beta-blocker medications provide a corrective measure to support cardiac remodeling and improve outcomes. Perhaps overwhelming hypoxia in multiple bilateral lung regions could foster diffuse hypoxic pulmonary vasoconstriction that in effect compromises adequate perfusion. In other words, with excessive hypoxia occurring diffusely throughout bilateral lung fields such as in COVID-19, HAPE and other conditions - the innate physiologic mechanism - may not be the most efficacious process to mitigate these aberrant, extreme and unusual conditions. Moreover, if unbridled, the homeostatic process may potentially be deleterious in overall outcomes in these particular situations. Therefore, overriding vasoconstriction with vasodilator therapy may be appropriate in order to sustain perfusion against a physiologic response that may be counterproductive in these isolated circumstances. And in fact, vasodilation with calcium channel blockers was found to be associated with improved survival in patients with COVID-19 [5].

**Figure 4:**
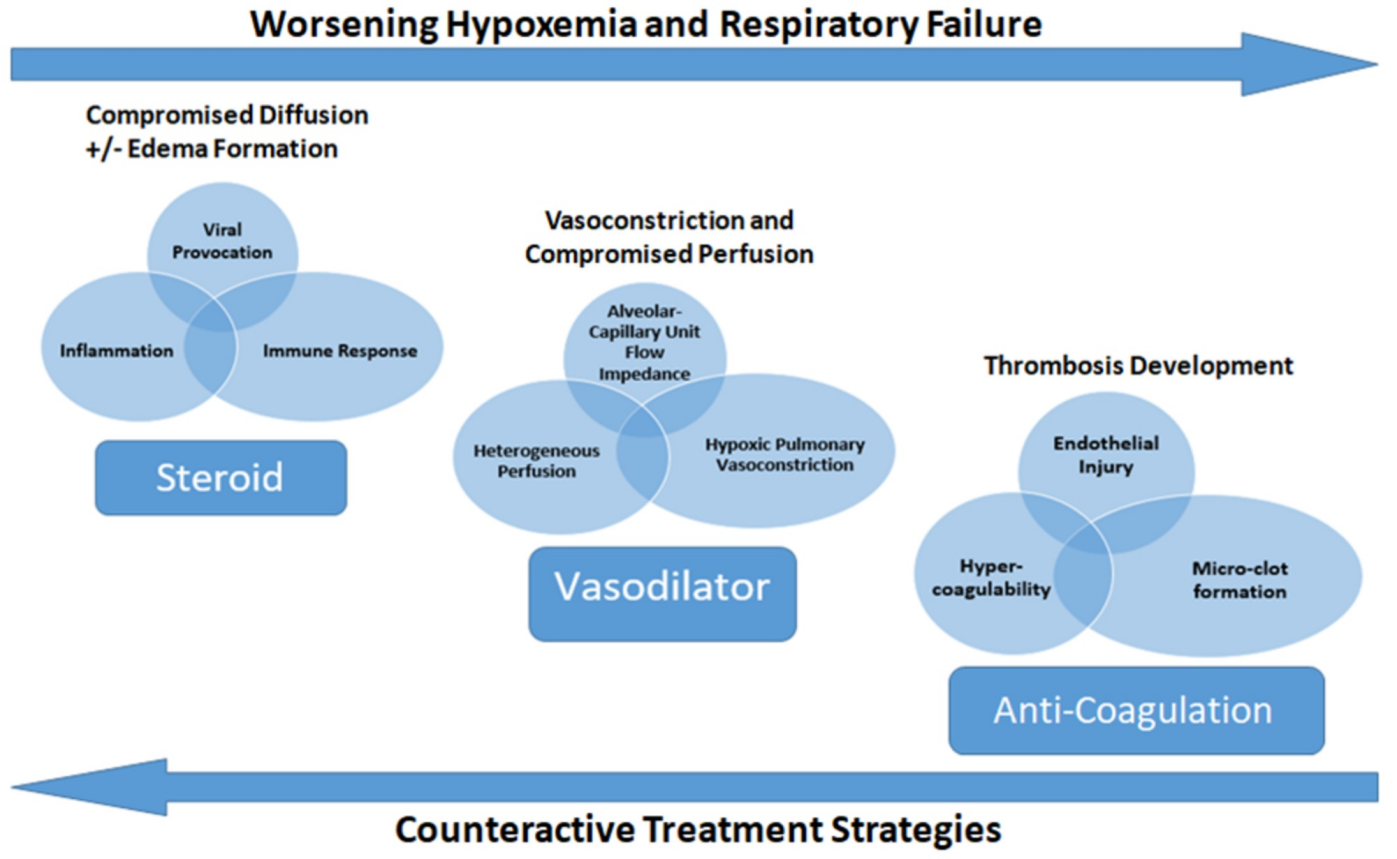
Pathophysiologic schematic of hypoxemia progression, development of respiratory failure and possible treatment strategies in COVID-19. Circles and ovals indicate pathogenic challenges. Rectangles indicate corresponding treatment strategies.

What methods may be utilized to improve perfusion? Well anti-coagulation is one that is already employed and a longer duration of use was associated with a reduced risk of mortality [16]. Corticosteroid use has been implemented. Perhaps efficacy may be augmented and/or synergistic in combination regimens. However, a wholly under-recognized strategy is direct vasodilation. Agents that vasodilate may promote perfusion as well. In fact, amlodipine and nifedipine were found to be associated with improved mortality and decreased risk of intubation in elderly hospitalized patients with COVID-19 as mentioned earlier [5]. Hypoxic pulmonary vasoconstriction can be alleviated. The tissue hypoxia and angiogenic response described above can be offset. Perhaps combining all three strategies, each providing a different and unique mechanism, may be most effective. Anti-coagulation, steroids and vasodilation agents could be a triple bundle that may improve overall outcomes (Figure 4).

Medications utilized to achieve vasodilation in HAPE have been previously suggested and include acetazolamide, CCBs, and phosphodiesterase inhibitors. Interestingly, aminophylline achieved similar results with increased oxygen levels with an even better effect than nifedipine in patients with HAPE [17]. Aminophylline may be an additional therapeutic agent with potential utility in COVID-19.

Cofactors can also play a role pulmonary vascular responses to hypoxia. Patient B was iron deficient and many other patients at the community hospital where both patients were seen do have iron deficiency as well. Iron status on its own modifies pulmonary vascular responses to hypoxia [18]. Further research into this may investigate if patients with iron deficiency may be at higher risk, plus, if intravenous iron infusion may be beneficial and improve outcomes in COVID-19.

Obviously viral triggered pathophysiologic processes and sequelae generating hypoxemia and tissue hypoxia are innately different from hypoxia at high altitude. However with analytical contemplation it is still possible to learn from various lung conditions where hypoxia is apparent. Comparing and contrasting is a learning method that can be utilized to decipher unique and similar aspects between separate conditions. Actual clinical data and findings may drive a search for effective clinical pathophysiologic models and treatment methods.

Compromise of micro-perfusion in various other pulmonary conditions should be considered including but not limited to Influenza, sickle cell disease, etc. Vasodilatation of pulmonary vasculature may be a pathway to improve perfusion and offset disease progression. It may further enhance the benefit of supportive ventilation interventions. Calcium channel blockers among other agents may be fit for this purpose. Yet, with further study, other vasodilatory agents such as those delivered continuously via a direct intravenous route may emerge as being even more efficacious.

Similar to the import placed on maintenance of preservation of blood flow via coronary arteries to allow for perfusion of cardiac muscle, preservation of perfusion via the pulmonary capillary unit may increasingly emerge as a key component in multiple respiratory conditions in order to maintain adequate oxygenation and capacity for respiratory function.

Interestingly, it has been demonstrated that improving the cardiac index in ARDS, as a result of a vasodilatation agent, can significantly improve the systemic oxygen delivery rate irrespective of an increased venous admixture [19]. Furthermore, in severe ARDS, elevation of pulmonary vascular resistance is a common finding and vasodilator therapy will lower capillary wedge pressure while increasing cardiac output and venous admixtures suggesting diffuse vasoconstriction is present therein [20]. Moreover, vascular occlusions identified in a majority of patients in that study correlated with increased post mortem counts of thrombi as well. This is reflective of thrombi mentioned above in HAPE and COVID-19.

Ultimately, the import of perfusion vis-a-vis ventilation may be underappreciated. Reexamining our focus and perspective on utilizing agents to improve the former may yield benefit in the treatment of multiple diffuse bilateral pulmonary conditions wherein hypoxia occurs.

## Conclusions

Altogether, this case study and literature review provides insight into the heterogeneous perfusion present in two hospitalized patients suffering from COVID-19 disease. The heterogeneous perfusion pattern is reflective of other conditions with hypoxia exposure including HAPE. Treatment strategies to improve perfusion were emphasized. These incorporate vasodilator agents in particular but also highlight the role of anti-coagulation to prevent and/or treat micro-clot formation. Micro-clot formation was also reflected to occur in both COVID-19 and HAPE. Together, hypoxia can be a driving force in both conditions experiencing heterogeneous perfusion patterns. Hypoxic pulmonary vasoconstriction may be aberrant when diffuse bilateral or multifocal pulmonary conditions and challenges exist. Reversing constriction with vasodilation agents may be beneficial. Improvement of hypoxia is advocated with the use of medications that promote micro-perfusion. A combination treatment strategy including anti-coagulation, steroids, and vasodilatory agents is suggested. The importance of promoting perfusion in the backdrop of ventilation may be underappreciated. Reexamining our focus and perspective on utilizing agents to improve the former may provide benefit in the treatment of multiple diffuse bilateral or multifocal pulmonary conditions wherein hypoxia transpires.
